# On Measuring miRNAs after Transient Transfection of Mimics or Antisense Inhibitors

**DOI:** 10.1371/journal.pone.0055214

**Published:** 2013-01-24

**Authors:** Daniel W. Thomson, Cameron P. Bracken, Jan M. Szubert, Gregory J. Goodall

**Affiliations:** 1 Centre for Cancer Biology, South Australia Pathology, Adelaide, South Australia, Australia; 2 Department of Medicine, University of Adelaide, Adelaide, South Australia, Australia; 3 School of Molecular and Biomedical Science, University of Adelaide, Adelaide, South Australia, Australia; German Cancer Research Center, Germany

## Abstract

The ability to alter microRNA (miRNA) abundance is crucial for studying miRNA function. To achieve this there is widespread use of both exogenous double-stranded miRNA mimics for transient over-expression, and single stranded antisense RNAs (antimiRs) for miRNA inhibition. The success of these manipulations is often assessed using qPCR, but this does not accurately report the level of functional miRNA. Here, we draw attention to this discrepancy, which is overlooked in many published reports. We measured the functionality of exogenous miRNA by comparing the total level of transfected miRNA in whole cell extracts to the level of miRNA bound to Argonaute following transfection and show that the supraphysiological levels of transfected miRNA frequently seen using qPCR do not represent the functional levels, because the majority of transfected RNA that is detected is vesicular and not accessible for loading into Argonaute as functionally active miRNAs. In the case of microRNA inhibition by transient transfection with antisense inhibitors, there is also the potential for discrepancy, because following cell lysis the abundant inhibitor levels from cellular vesicles can directly interfere with the PCR reaction used to measure miRNA level.

## Introduction

MicroRNAs are small endogenous RNA molecules that guide the RNA-protein complex, RISC (RNA induced silencing complex), to target sequences in mRNAs. The biosynthesis and functions of miRNAs have been reviewed recently [1]. RISC-loaded miRNAs bind in a sequence-specific manner to target mRNAs, initiating their repression through a combination of translational inhibition, RNA destabilisation (via de-capping and de-adenylation) or, albeit rarely in mammals, direct RISC-mediated mRNA cleavage [2], [3], [4], [5], [6], [7]. The majority of mRNA transcripts are subject to direct miRNA-mediated regulation, largely via interactions with target 3′ untranslated regions. Consequently, miRNAs are directly or indirectly involved in most biological processes and have been extensively implicated in such areas as development, immune regulation and cancer progression.

## Results and Discussion

**Figure 1:**
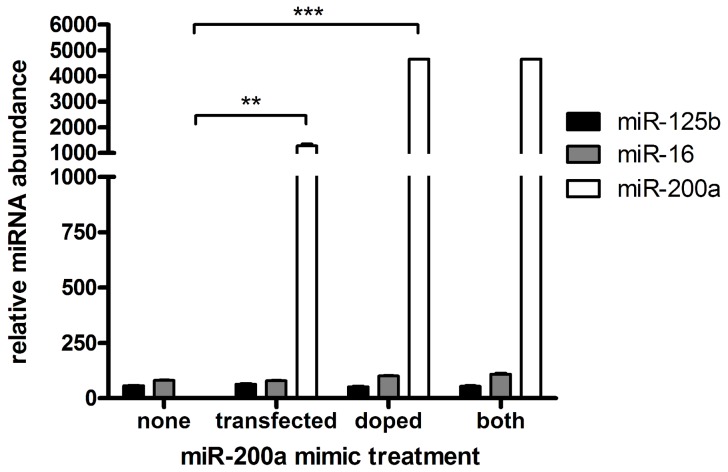
Measurement of miRNA by qRT-PCR after transient transfection with miRNA mimic. miR-200a, miR-125b and miR-16 levels were quantitated by qPCR following either transfection of the miR-200a mimic in MDA-MB-231 cells, following addition of the miRNA mimic post lysis (doping), or after both transfection and doping. Experiments were performed as biological triplicates with error bars depicting standard error of mean. Asterisks denote significance, *** p<0.001, ** p<0.01.

For a miRNA to be functional, it must be incorporated into RISC. While qPCR is a simple and commonly used method to measure the level of a miRNA, it does not distinguish between miRNAs in functional or non-functional pools. To assess whether the majority of transiently transfected miRNA resides in a functional location, we transfected miR-200a mimic into MDA-MB-231 cells, which have very little endogenous miR-200a, and measured the miR-200a level after 2 days by TaqMan qPCR assay or by immunoprecipitation with anti-Ago antibody followed by deep sequencing. Measurement of the transfected miRNA by qPCR indicated miR-200a was increased by >1000- fold, to a level vastly greater than the most abundant endogenous miRNAs, such as miR-125b and miR-16 (Fig. 1). However, we found that double-stranded miRNA mimics added to cell extracts post-lysis were also detected at high level by the qPCR (Fig. 1), demonstrating that qPCR amplification alone does not necessarily indicate functionality.

**Figure 2:**
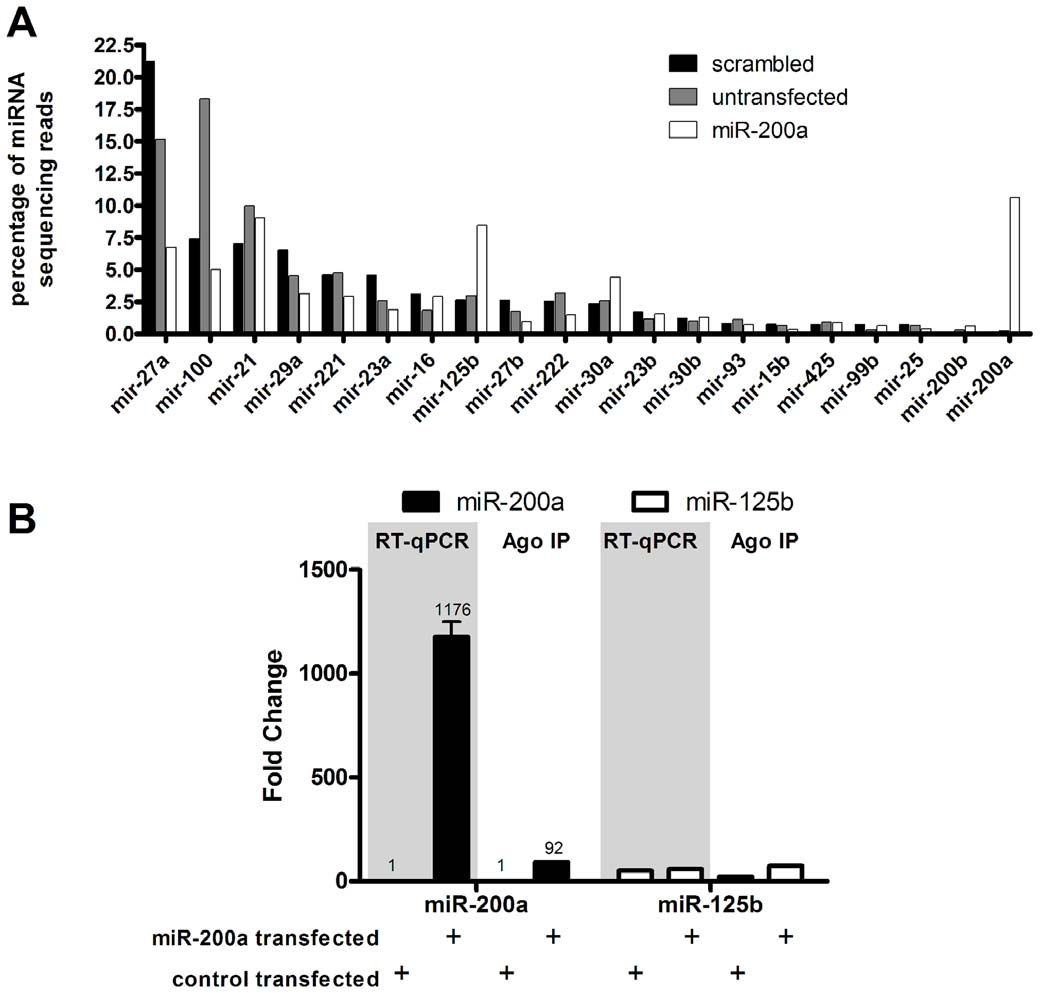
Quantitation of functional transfected miRNA mimic by deep sequencing of RNA from Argonaute immunoprecipitation. **A**) MDA-MB-231 cells were transfected with miR-200a, with scrambled control, or were untransfected, then subjected to UV-crosslinking and Argonaute immunoprecipitation followed by deep sequencing of the Argonaute-bound small RNA pool. The levels of miRNAs (x-axis) are represented as a percentage of total miRNA sequencing reads (y-axis). Similar results were obtained using transfection of miR-200b. **B**) miR-200a and miR-125b were measured by qPCR from whole cell lysate or by deep sequencing AGO-immunoprecipites from control and miR-200a-transfected MDA-MB-231 cells. In each case the fold change is calculated by comparing to basal miR-200a levels.

To measure the level of functional miRNA in a manner that avoids detecting miRNA mimic trapped in non-functional locations, we immunoprecipitated UV cross-linked RISC from control and transfected cells and measured the amount of RISC-associated miR-200a by deep sequencing of the miRNA-sized RNA fraction in the immunoprecipitate. This revealed that the amount of RISC-associated miR-200a in the transfected cells was approximately equal to the level of other abundant miRNAs (Fig. 2A). This is proportionally much less than the level of miR-200a measured by qPCR (Fig. 1, Fig. 2B), indicating most of the transfected miRNA mimic is not bound to Argonaute and consequently is not functional. Similar results were obtained following transfection of a different miRNA, miR-200b (data not shown). Thus, although qPCR is a valid technique to measure total miRNA amount, this can be very different from the amount of functional miRNA.

**Figure 3:**
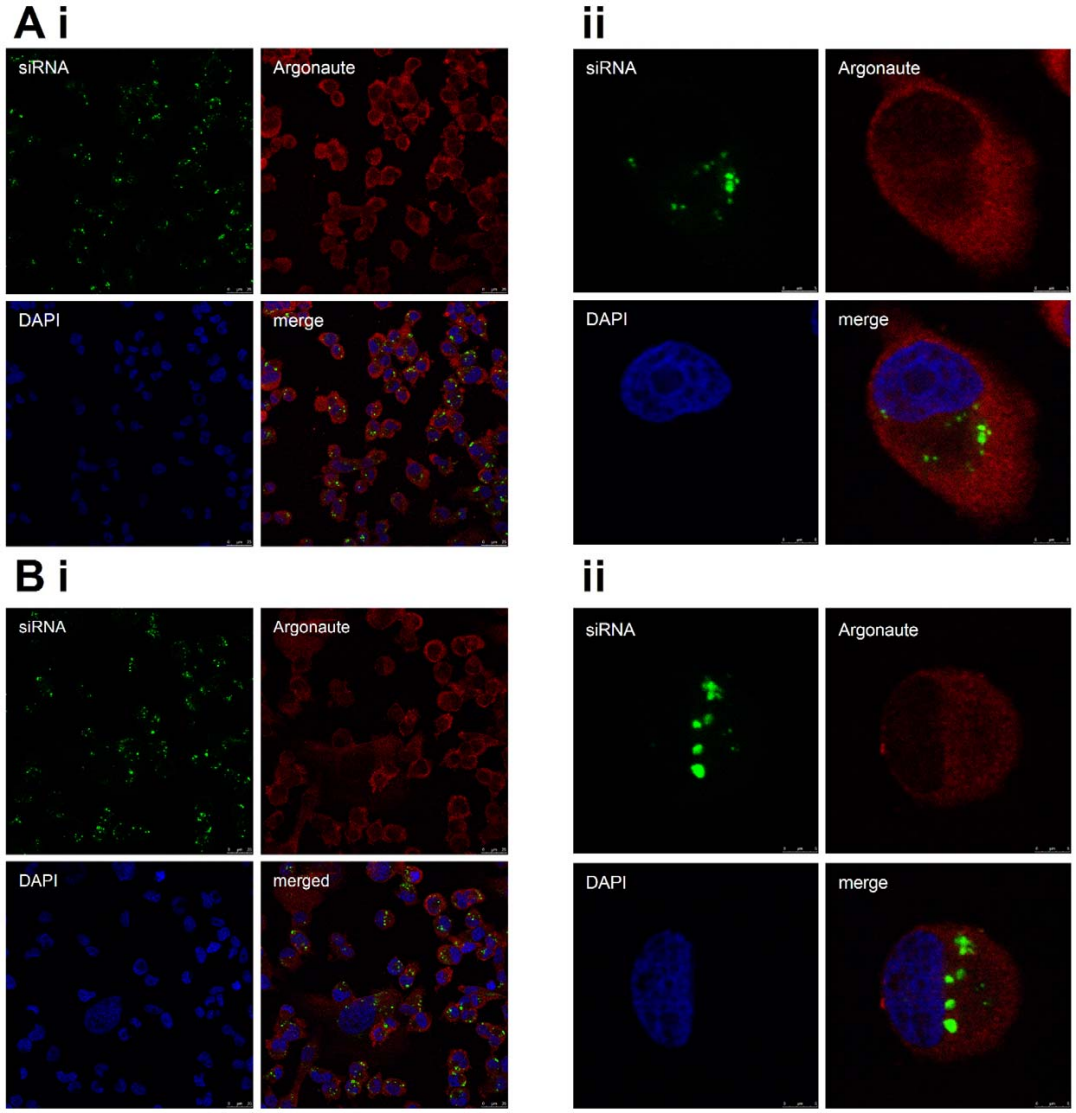
Transfected siRNAs show little co-localisation with Argonaute. MDA-MB-231 cells were visualised by fluorescence microscopy showing transfected fluorescent siRNA (green), endogenous Argonaute (red, visualised by immunofluorescence using 2A8 antibody) and nuclei (DAPI, blue). Cells were transfected using either A) Lipofectamine 2000 (Invitrogen) or B) HiPerfect (Qiagen). Representative images are shown.

**Figure 4:**
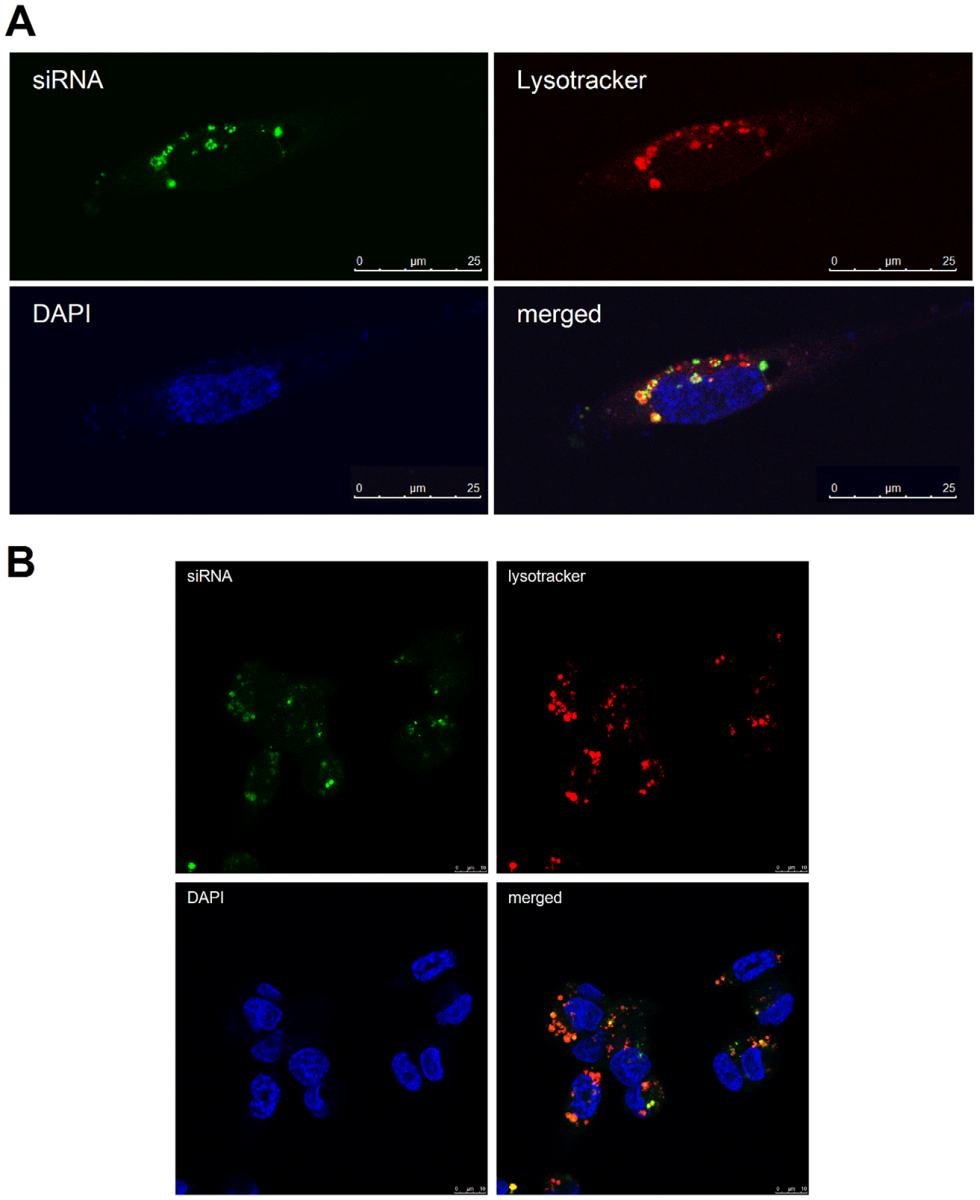
Transfected siRNAs localise with and adjacent to lysosomes. MDA-MB-231 cells were visualised by fluorescence microscopy showing transfected fluorescent siRNA (green), lysosomes (lysotracker, red) and nuclei (DAPI, blue). Cells were transfected with either **A**) HiPerfect (Qiagen) or **B**) RNAi-Max (Invitrogen).

Given the majority of miRNA mimic detected by qPCR did not represent the active Argonaute-bound population, we determined its sub-cellular localisation by transfecting a fluorescent siRNA and examining the transfected cells by fluorescence microscopy. The majority of the siRNA did not co-localise with Argonaute (Fig. 3A; Fig. 3B), which is consistent with earlier reports of transfected siRNA localising in large cytoplasmic aggregates that are distinct from the GW bodies that are known for their role in RNA silencing [8]. Instead the vast majority of miRNA transfected with either HiPerfect, (Fig. 4a), RNAi-Max (Fig. 4b) or Lipofectamine 2000 (data not shown) localised with or adjacent to lysosomes, matching earlier reports of lipid-based siRNA transfection [9], [10]. Therefore, the high level of transfected miRNA detected by qPCR is largely attributable to their retention within vesicles and subsequent amplification by qPCR following lysis. Hence, the use of qPCR to measure a miRNA after transient transfection can give the false impression that the miRNA is at massively non-physiological level, whereas the amount of miRNA bound to Argonaute may indeed be appropriately physiological. On the other hand, it is conceivable that an inefficient transfection that results in just a small proportion of cells being transfected could appear to produce an adequate level of miRNA, if measured by qPCR. It is more appropriate to use an assay of miRNA function to verify the effectiveness of the transfection.

**Figure 5:**
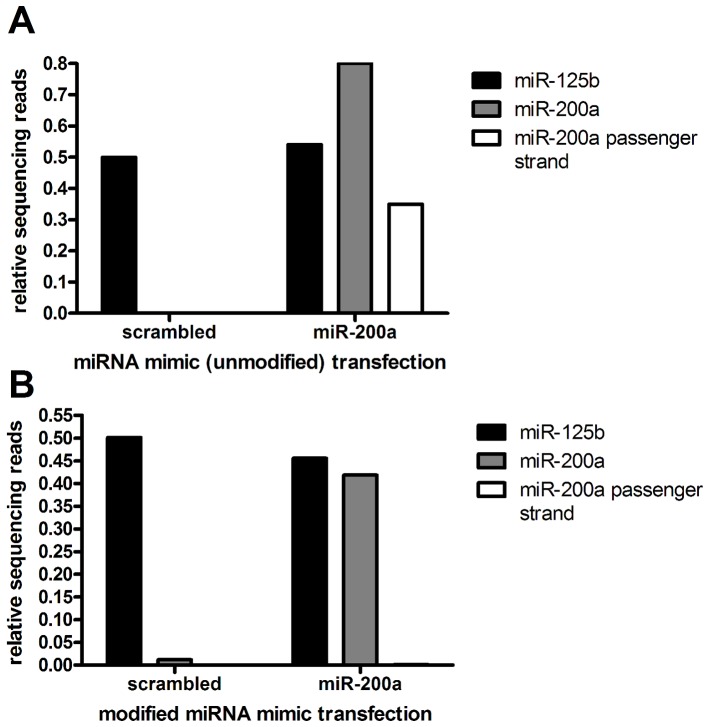
The passenger strand of an unmodified miRNA mimic is incorporated into RISC. MDA-MB-231 cells were transfected with 60 nM scrambled or miR-200a (double-stranded) miRNA mimic. Samples were then subjected to Argonaute immunoprecipitation and deep sequencing. Sequence read numbers are shown relative to miR-125b. **A**) Unmodified miRNA mimics showed significant co-precipitation of the passenger strand as well as the guide miR-200a strand. **B**) A miRNA mimic modified to circumvent this problem (Ambion) successfully eliminates incorporation of the passenger strand.

Of additional interest to users of miRNA mimics for transient transfection, we were able to confirm from our sequencing of the Argonaute-bound pool of small RNAs, that while a miRNA mimic with unmodified passenger strand results in abundant incorporation of the passenger strand into RISC (Fig. 5A), raising the potential for extensive off-target effects, a mimic that is modified to limit the incorporation of the passenger strand into RISC does indeed achieve this (Fig. 5B). Although the merits of modified mimics have been previously recognised, published evidence for this is limited to date and has been based largely on reporter assays comparing the response of reporters that harbour a target site for either the siRNA sense strand or passenger strand (http://products.invitrogen.com/ivgn/product/AM17100). Our observation provides additional support for the lack of incorporation of modified passenger strand.

**Figure 6:**
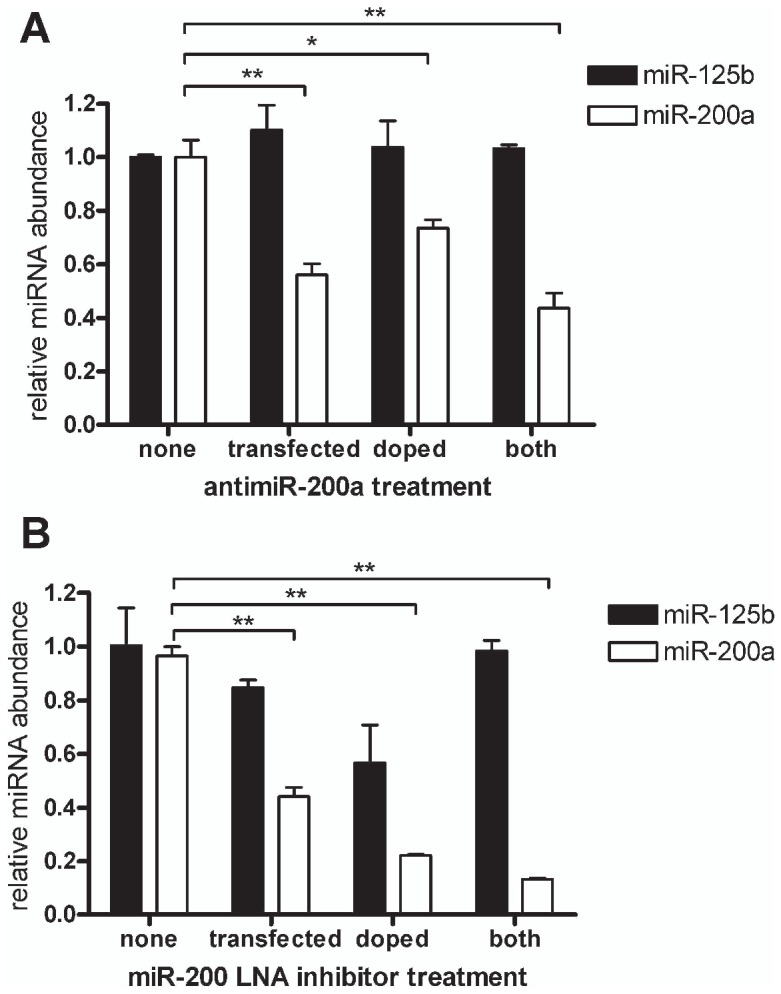
Measurement of miRNA by qPCR after antisense inhibitor transfection does not reliably measure the level of the cellular miRNA. qPCR of miR-200a and miR-125b in MCF7 cells following transfection with antisense miR-200a (antisense inhibitor), or after its addition post lysis (doping), or both. miR-200a antisense inhibitors were either modified as A) 2′-O-methyl oligonucleotides or B) LNA-oligonucleotides. Experiments were performed in biological triplicate with error bars depicting standard error of mean. Asterisks denote significance, *** p<0.001, ** p<0.01, * p<0.05.

qPCR is also sometimes used to verify the inhibition of a miRNA by transiently transfected antisense inhibitor, but this can also be problematic because the antisense inhibitor can directly inhibit the qPCR reaction. For example, in an experiment where transfection of miR-200a antisense inhibitor into MCF7 cells produced an apparent ∼50% decrease in miR-200a levels as measured by qPCR (Fig. 6A), we found that much of the apparent decrease in miRNA was attributable to the suppressive effect of antisense inhibitor on the PCR reaction itself. This was revealed by the addition of the same amount of antisense inhibitor directly to the cells after lysis by TRIzol, but prior to RNA extraction, which appeared to give a similar decrease in the level of miR-200a as measured by qPCR. Coupled with the fact that most of the transfected oligonucleotide is located in vesicles, this indicates that the qPCR may be largely measuring the inhibitory effect of the vesicle-associated antisense inhibitors on the qPCR, rather than its antisense activities within cells. We note that both 2′-O-Methyl and LNA (locked nucleic acid) miRNA inhibitors are similarly subject to this phenomenon (Fig. 6A, B). This complements previous observations that the LNA:miRNA complex interferes with the binding of the Northern blot probe when measuring miRNA inhibition by Northern blot [11].

Whilst miRNA mimics and antisense inhibitors are valuable tools, our observations indicate caveats to the analysis of miRNA and antisense inhibitor transfection that are apparently not universally appreciated, leading to the surprisingly frequent use in the literature (examples available on request) of qPCR for mRNA measurement when a readout of function would be more appropriate. Better options are the use of a miRNA reporter (such as luciferase or a fluorescent protein under the control of miRNA target sites) to report the relative functional level of a miRNA, or measurement of the miRNA level following Argonaute immunoprecipitation.

## Materials and Methods

### Quantitative PCR following miRNA mimic transfection or addition post lysis

60 nM miR-200a mimic (Ambion or Genepharma) was transfected into MDA-MB-231 cells using Lipofectamine 2000 transfection reagent (Invitrogen) or Lipofectamine RNAiMAX (Invitrogen) compared to a scrambled control miRNA. Cells were incubated at 37°C, 5% CO_2_ for 48 h and harvested with TRIzol reagent (Invitrogen). Prior to RNA extraction samples were doped (addition without transfection) with the equivalent amounts of miR-200a mimic. qRT-PCR was performed using MultiScribe RT and TaqMan probes (Applied Biosystems) for hsa-miR-200a, hsa-miR-16 and hsa-miR-125b. Unmodified miRNA mimics were obtained from GenePharma and proprietary modified miRNAs (designed for selective incorporation of the guide strand into RISC) are from Ambion.

### Argonaute: miRNA immunoprecipitation

MDA-MB-231 cells were grown in 20×10 cm dishes and transfected with 60 nM miRNA mimic (Ambion/GenePharma) using HiPerfect transfection reagent (Qiagen). 24 h later, cells were suspended in ice-cold PBS by scraping and subjected to UV crosslinking at 254 nM (Stratalinker). Cell pellets were lysed (0.1% SDS, 0.5% deoxycholate, 0.5% NP-40 with protease inhibitors, Roche) for 10 mins on ice followed by RQ1 DNAse (Promega) at 37°C for 15 mins with shaking. RNAse A/T1 (Ambion) was then added for a further 8 mins, prior to the addition of EDTA (30 mM). Pellets were then spun (30,000 rpm) and the lysate subjected to immunoprecipitation for 2 h with a pan-anti-Ago antibody (2A8, kind gift of Zissimos Mourelatos) conjugated to protein-A dynabeads (Invitrogen) using bridging rabbit anti-mouse IgG (Jackson Immunolabs). Pellets were then successively washed (0.1% SDS, 0.5% deoxycholate, 0.5% NP40 in 1× PBS; 0.1% SDS, 0.5% deoxycholate, 0.5% NP40 in 5× PBS; 50 mM Tris pH 7.5, 10 mM MgCl_2_, 0.5% NP40) and on-bead phosphatase treatment performed for 30 mins with antarctic phosphatase (New England Biolabs) in the presence of superasin RNAse inhibitor (Ambion). The 3′ RNA linker (CAGACGACGAGCGGG) was labeled with P^32^ using T4-PNK (NEB) and ligated on-bead for 1 h at 16°C with T4 RNA ligase (Fermentas). Beads were then washed as previous and treated with PNK to ligate the 5′ RNA linker (AGGGAGGACGAUGCGGxxxG, with “X” representing different nucleotides for barcoding). Beads were resuspended in 4x LDS Novex loading buffer with 4% B-mercaptoethanol, incubated at 70°C for 10 mins and the supernatant loaded on Novex NuPAGE 4-12% Bis-Tris acrylamide gels (Biorad). After running, the Ago-RNA complexes were then transferred to nitrocellulose and exposed to film at −80°C for 3 days. Complexes running at ∼110 kDa were then excised with a scalpel and resuspended (100 mM Tris pH 7.5, 50 mM NaCl, 10 mM EDTA, 4 mg/ml proteinase K) for 20 mins at 37°C. The sample was incubated for an additional 20 minutes in the presence of 3.5 M urea and RNA isolated by a phenol-chloroform extraction. Sample was then run on a 10% denaturing (1∶19) polyacrylamide gel and exposed to film with an intensifying screen at −80°C for 5 d. A thin band corresponding to labelled miRNAs was excised, crushed and eluted at 37°C for 1 h (1 M NaOAc, pH 5.2, 1 mM EDTA). RNA was then precipitated overnight with ethanol, centrifuged and dried. RNA was then resuspended in 8 µl H_2_O, primer added (TCCCGCTCGTCGTCTG) and reverse transcription performed using SuperScriptIII (Invitrogen). PCR was then performed with the above primer and an additional primer (ACGGAGGACGATGCGG) for 25 cycles. PCR product was then run on a 10% native (1∶29) polyacrylamide gel, stained with Sybr Gold (Qiagen) and bands excised over a UV light box. The DNA was then precipitated using isopropanol and a final 10 cycle PCR performed with the following primers:

AATGATACGGCGACCACCGACTATGGATACTTAGTCAGGGAGGACGATGCGG, CAAGCAGAAGACGGCATACGATCCCGCTCGTCGTCTG. Reactions were then run on 2% metaphor agarose/TBE gels and bands (∼115 bp) excised corresponding to the linker sequence + miRNA CLIP tag. Samples were finally purified using quick-spin columns (Qiagen) and subjected to Illumina GAII 35 bp read-length deep sequencing (Geneworks).

### Bioinformatics

Using an in house Perl script, Illumina GAII 36 bp reads were first filtered for average quality and for homopolymeric tracts exceeding 12 nt, trimmed of linker sequence fragments and separated by barcode. The program bowtie [12] was used to align resulting 17 to 30 nt reads to the human genome (NCBI36/Hg18 downloaded from UCSC – http://genome.ucsc.edu), allowing a maximum of one mismatch and permitting up to 15 map locations. Reads with the same sequence were consolidated into single BED format file entries, specifying the accumulated number of tags and made available for display and analysis [12].

### Fluorescent oligonucleotide transfection, Lysotracker transfection, and immunofluorescence microscopy

MDA-MB-231 cells were transfected with 60 nM FAM labelled negative control duplex oligonucleotide (Genepharma) on chamber-slides coated with fibronectin and cultured in DMEM/20% FCS. Following a 48 h incubation at 37°C, 5% CO_2_, 5 nM Lysotracker red (Invitrogen) was applied to cells for 30 min. Cells were washed in 37°C PBS for 5 min then fixed in 37°C 4% PFA, pH 9.3 for 5 min. Cells were washed twice in PBS then blocked in 1% BSA/0.3% Triton X-100 for 20 min at RT. After three washes in PBS cells were permeabilised in 0.3% Triton/PBS. Pan-anti-Ago antibody (2A8, Zissimos Mourelatos) was applied at 1 in 250 for 1 h at RT. After 2x PBS washes, secondary antibody Alexa Fluor 594 goat, anti-mouse IgG2a (Invitrogen, product No A-11005) was applied for 1 h at RT. DAPI was applied for 5 min then slides were washed twice in PBS and mounted in DAKO aqueous fluorescent mounting medium. Cells were imaged using a Leica SP5 spectral scanning confocal microscope.

### miRNA inhibitor doping experiment

60 nM antisense inhibitor miR-200a or scrambled control antisense inhibitor (Dharmacon) was transfected into MCF7 cells using Lipofectamine 2000 or Lipofectamine RNAi max (Invitrogen). Cells were incubated at 37°C, 5% CO_2_ for 48 h. Cells were harvested with TRIzol reagent (Invitrogen). Prior to continuing with RNA extraction samples were doped (added after cell lysis) with the equivalent amount of miR-200a antisense inhibitor. qRT-PCR was performed using Taqman probes (Applied Bioscience) for miR-200a and miR-125b. Experiments were also performed with 60 nM miR-200 or control LNA inhibitor (Integrated DNA Technologies).
